# A protein-centric approach for the identification of folate enzymes from the malarial parasite, *Plasmodium falciparum*, using OFFGEL™ solution-based isoelectric focussing and mass spectrometry

**DOI:** 10.1186/1475-2875-9-286

**Published:** 2010-10-18

**Authors:** Ronan DM O'Cualain, John E Hyde, Paul FG Sims

**Affiliations:** 1 Manchester Interdisciplinary Biocentre, Faculty of Life Sciences, University of Manchester, 131 Princess Street, Manchester M1 7DN, UK

## Abstract

**Background:**

Plasmodium species are difficult to study using proteomic technology because they contain large amounts of haemoglobin-derived products (HDP), generated by parasite breakdown of host haemoglobin. HDP are known to interfere with isoelectric focussing, a cornerstone of fractionation strategies for the identification of proteins by mass spectrometry. In addition to the challenge presented by this material, as in most proteomes, there exists in this parasite a considerable dynamic range between proteins of high and low abundance. The enzymes of the folate pathway, a proven and widely used drug target, are included in the latter class.

**Methods:**

This report describes a work-flow utilizing a parasite-specific extraction protocol that minimizes release of HDP into the lysate, followed by in-solution based OFFGEL™ electrophoresis at the protein level, trypsin digestion and mass spectrometric analysis.

**Results:**

It is demonstrated that, by removing HDP from parasite lysates, OFFGEL™-mediated protein separation is able to deliver reduced complexity protein fractions. Importantly, proteins with similar and predictable physical properties are sharply focussed within such fractions.

**Conclusions:**

By following this novel workflow, data have been obtained which allow the unequivocal experimental identification by mass spectrometry of four of the six proteins involved in folate biosynthesis and recycling.

## Background

The rapid and sensitive characterization of the *Plasmodium falciparum *proteome is now potentially possible due to advances in the field of mass spectrometry and associated technologies, including the sequencing of its genome [1]. Previous work from this laboratory described the fractionation of parasite lysates using a solution-based electrophoretic strategy [2]. The application of this strategy resulted in the successful identification of serine hydroxymethyltransferase (SHMT) by mass spectrometry. The reliable and reproducible identification of other folate enzymes has not, however, been possible so far, mainly due to the problem of haemozoin, the biomineralized end product of haem sequestration [3], and haemoglobin-derived products (HDP). HDP result from the release of haem groups and other protein degradation products by the parasite's digestion of the haemoglobin within infected red blood cells [4,5]. The problem posed by HDP is important since they are believed to constitute a significant proportion of the biomass of the parasite [6], and the most widely-used protocol for protein extraction in *P. falciparum *[7] destabilizes the HDP complexes present in parasite cells. In this urea-thiourea containing extraction protocol, the food vacuole membrane within which HDP reside in the parasite is thought to be solubilized, resulting in large amounts of contaminating HDP of varying molecular weights being released into the lysate and hindering downstream applications.

The enzymes of the *P. falciparum *folate biosynthetic pathway and associated thymidylate cycle comprise GTP cyclohydrolase I (GTPCI; EC 3.5.4.16), 6-pyruvoyltetrahydropterin synthase (PTPS; EC 4.2.3.12), bifunctional 6-hydroxymethyldihydropterin pyrophosphokinase-dihydropteroate synthase (HPPK-DHPS; EC 2.7.6.3 and EC 2.1.5.15 respectively), bifunctional dihydrofolate synthase-folylpolyglutamate synthase (DHFS-FPGS; EC 6.3.2.12 and EC 6.3.2.17 respectively), serine hydroxymethyltransferase (SHMT; EC 2.1.2.1) and bifunctional dihydrofolate reductase-thymidylate synthase (DHFR-TS; EC 2.1.1.45 and EC 1.5.1.3 respectively) [8]. Drugs targeting the parasite folate pathway constitute a major line of defence against the widespread strains of chloroquine resistant *P. falciparum *[9]. A work-flow that allows the reproducible and facile identification of these enzymes by mass spectrometry would be valuable as it would then allow the design of experiments such as quantitation of enzyme levels in response to drug intervention.

OFFGEL™ electrophoresis is a relatively new development in proteomic research where zwitterionic molecules are focussed in solution according to their isoelectric point. The separated components are recovered in liquid fractions which greatly facilitates downstream processing, allowing multi-dimensional separations of complex samples. Focussing in this way allows for relatively large-scale separations. Previously described applications of OFFGEL™ technology have employed solution-based fractionation at the peptide level and, in this way, it has been possible to rapidly identify proteins in complex mixtures using a shotgun approach with pre-digested protein fragments [10-12]. This report describes the use of OFFGEL™ electrophoresis at the protein level and illustrates the advantages it too offers for proteomic research in general, particularly by providing access to low-abundance proteins.

With reference to plasmodial proteomics, our work-flow overcomes the issue of HDP in parasite lysates and the wide dynamic range of the parasite proteome, allowing identification of the low-abundance proteins of the folate pathway by mass spectrometry. A key step in facilitating this has been the development of a cell lysis protocol that generates extracts unencumbered with significant levels of HDP. Here, it is shown that this malaria-specific step can be a valuable complement to the generally applicable, and as yet under-explored, capability of OFFGEL™ electrophoresis for protein separation. In previous work from this laboratory, it was demonstrated how solution-phase isoelectric focussing (IEF) of proteins in complex lysates could contribute to their identification by mass spectrometry [2]. Here, this approach is refined and improved, introducing the much greater level of fractionation that is afforded by the OFFGEL™ technology. Combined into the new workflow, these developments have enabled even deeper mining of the *P. falciparum *proteome, such that, for the first time, proteins making up the majority of the folate pathway have been confidently identified.

## Methods

### Culture of *P. falciparum *and parasite sampling

*Plasmodium falciparum *K1 parasite lines were grown at 37°C as described [13,14] in washed O^+ ^erythrocytes in RPMI 1640 medium (Invitrogen), supplemented with 0.5% bovine serum albumin, 5 μg/mL hypoxanthine and 50 μg/mL gentamicin sulphate (all from Sigma Aldrich). Synchronization was performed as described [15] using the sorbitol method. Briefly, parasites were cultured to about 1% parasitaemia and a high proportion of ring-forms, the cultures were centrifuged at 1,000 *g *for 5 min, and the supernatant discarded. The cells were resuspended in sorbitol solution (10% [v/v]) and allowed to stand for 5 min at 22°C. The cells were then washed twice in RPMI medium and finally resuspended in RPMI. This process was repeated after 32 hrs to ensure a high level of synchrony. Using this culture as the inoculum, 16 × 150 cm^2 ^flasks were set up (Corning cat. # 10-126-32) at 2% haematocrit (1 mL blood and 50 mL medium per flask) and at 0.1% parasitaemia on the first day (d 0). When parasitaemia had increased to about 5% (d 3), the haematocrit was increased to 8% by adding freshly washed red blood cells, and the medium was changed. The parasitaemia and culture progression was monitored every 24 hours by Giemsa staining. When parasitaemia had reached 8% (d 6) and parasites were 30 hrs into their cycle, the culture was collected. Each flask was estimated to contain approximately 6 × 10^9 ^parasites.

### Sample preparation

*Plasmodium falciparum *parasites were released from infected erythrocytes by lysis in 0.05% saponin in phosphate-buffered saline (PBS) for 5 min at 4°C and then centrifuged at 800 *g *and 4°C for 5 min. The parasite pellets were repeatedly washed in ice-cold PBS and spun at 1,000 *g *for 2 min until contaminating blood cell lysis products, medium and saponin were no longer apparent in the PBS after resuspension. Pellets were weighed, snap-frozen in liquid nitrogen and stored at -80°C. The freeze-thaw extraction for pre-fractionation was conducted as follows. For each 100 μg of parasite pellet, 2.5 mL of deionized water and 5 μL of protease inhibitor cocktail (Sigma Protease Inhibitor Cocktail, P8340) was added. The pellet was resuspended by carefully pumping the suspension into and out of the pipette tip until it had been completely dispersed. The suspension was then frozen in liquid nitrogen for 10 min, thawed in a water bath at 37°C, and vortexed for 30 sec at room temperature followed by 30 sec cooling in an ice water bath for a total of 3 min (3 cycles of vortexing). This comprised one freeze-thaw cycle, and this process was then repeated for a total of 5 cycles. Insoluble material was pelleted and the supernatant collected and clarified by centrifugation three times at 12,000 *g *for 20 min at 4°C. Proteins in the supernatant were prepared for OFFGEL™ IEF using acetone precipitation, whereby 5 volumes of ice-cold acetone were added to the solution and held at -20°C overnight. Protein was pelleted by centrifugation at 12,000 *g *for 20 min, the pellets were air-dried and resuspended in OFFGEL™ buffer prior to IEF. Protein concentration was measured using the 2D Quant kit (GE Healthcare) with bovine serum albumin (BSA) as the standard.

During the early stages of this work, a urea/thiourea extraction was also utilized. Parasite pellets were extracted in lysis buffer (9.5 M urea (Serva)/2.0 M thiourea/1% dithiothreitol (DTT)/2.5 mM ethylenediaminetetraacetic acid (EDTA)/2.5 mM ethyleneglycoltetraacetic acid (EGTA; all from Sigma Aldrich)). Pellets were resuspended in lysis buffer and vortexed for 30 sec at room temperature followed by 30 sec cooling in an ice water bath for a total of 10 min (5 min vortexing). The vortexed pellet was allowed to stand at 22°C for 10 min and insoluble material was pelleted and the supernatant collected and clarified by centrifugation three times at 12,000 *g *for 20 min.

### Solution-based isoelectric focussing using the Agilent 3100 OFFGEL™ fractionator

Freeze-thaw extracts were separated using the Agilent 3100 OFFGEL™ fractionator. For fractionation using the 12 fraction frame, acetone precipitated pellets were reconstituted in 1.8 mL of OFFGEL™ buffer (7 M urea (Serva)/2 M thiourea/1% DTT/10% glycerol (all Sigma Aldrich)/1.0% (v/v) IPG buffer pH 3-10 (GE Healthcare). Protein was applied for fractionation at a concentration of approx. 600 μg/mL (total protein load was approx. 1 mg). Protein extract (150 μL) was loaded into each fraction. The samples were focussed using an optimized focussing protocol whereby the voltage was gradually increased over four hours from 500 V to 1000 V before a final limiting voltage of 8000 V was applied until 50 kV-h had been achieved. A maximum current of 50 μA was applied throughout the focussing stage. Temperature was stabilized to 22°C during the fractionation. Protein fractions were collected and alkylated at this stage with the addition of 1 M iodoacetamide to a final concentration of 50 mM and incubation at 22°C in darkness for 20 min. The 12 fractions were desalted using acetone precipitation as described above, with one exception; the samples were first diluted with two volumes of de-ionized water before five volumes of ice-cold acetone was added.

### Electrophoretic separation

For protein identification by mass spectrometry, the larger format Bio-Rad Protean II XL system was used (20 × 20 cm gel size), while for identification by antibody, the minigel format Bio-Rad Protean II system (7 × 10 cm gel size) was used. Acetone precipitated protein fractions, separated using the OFFGEL™ system, were reconstituted in Laemmli sample buffer (60 mM Tris-HCl pH 6.8, 2% w/v SDS, 3% v/v mercaptoethanol, 10% glycerol and a trace of bromophenol blue). Unstained Bio-Rad Precision Plus protein standard molecular weight (MW) marker was used to estimate molecular weight. The samples and MW marker were heated to 95°C for 5 min and then applied to a 1 mm thick 4% stacking, 12.5% resolving gel (prepared using the Bio-Rad Protean II XL system with overnight gel curing) for SDS-PAGE according to Laemmli [16]. Electrophoresis was carried out with a first phase current of 25 mA/gel for 1 hr until the proteins had entered the gel, followed by a second phase current of 35 mA/gel and a third phase maximum of 10 W/gel for the last hour until the dye front was approximately 5 mm from the bottom of the gel. The temperature was controlled to 18°C during electrophoresis using a Multitemp III thermostatic circulator (GE Healthcare). The small format gels were run at 200 V until the dye front was approximately 5 mm from the bottom. Proteins were stained using Coomassie Brilliant Blue (0.1% Coomassie G-250, 10% ammonium sulphate, 20% methanol, 3% phosphoric acid) and destained in 50% methanol, 8.75% acetic acid, 41.25% water.

### Western blot identification of GTPCI and DHFS-FPGS

The protein bands on gels were blotted onto a nitrocellulose membrane (Schleicher and Schuell) by electrophoresis using a transfer buffer of 200 mM glycine, 25 mM Tris-HCl pH 8.0, 20% methanol and 0.1% SDS. Proteins were transferred at 200 mA for 2 hours. The membranes were then blocked by treatment at room temperature with 1% fat-free dry milk in TBST (10 mM Tris HCl pH 8.0, 150 mM NaCl and 0.05% Tween 20) for 30 min. They were then incubated with a 1:500 dilution of the appropriate polyclonal chicken IgY antibody (raised against a recombinant form of the relevant protein expressed in *E. coli*; Eurogentec) in TBST to detect either GTPCI or DHFS-FPGS. Anti-PTPS was used as a positive control. After washing with TBST, the membranes were incubated with 1:5000-diluted alkaline phosphatase-conjugated mouse anti-chicken IgG (Promega). The bound secondary antibodies were detected with BCIP/NBT alkaline phosphatase substrate in 100 mM Tris-HCl pH 9.5, 100 mM NaCl, and 5 mM MgCl_2_. Once the colour had reached the desired intensity, the reaction was stopped by rinsing the membrane in deionized water for several minutes.

### In-gel trypsin digestion

For MS analysis, gel bands were sliced into small fragments approximately 1 mm^3 ^in size and were dehydrated and destained by two successive cycles of incubation in 50% acetonitrile, followed by 25 mM ammonium bicarbonate, each treatment for 15 min at 22°C, and then the gel pieces were dried using a vacuum centrifuge. The proteins were digested overnight at 37°C in 20 μL of sequencing grade trypsin (Promega, 1 μg in 100 μL of 50 mM ammonium bicarbonate). The resulting tryptic peptides were extracted with two incubations in 30 μL of 70% acetonitrile for 30 min each, followed by 90% acetonitrile for 15 min and finally 50 mM ammonium bicarbonate for 15 min. The supernatants were pooled and dried to near-dryness in a vacuum centrifuge. Sample loss and plasticizer contamination of mass spectra were minimized with the use of siliconized microfuge tubes/pipette tips and Teflon coated storage bottles.

### Mass spectrometric analyses

Mass spectrometric analyses were carried out by nanoflow-liquid chromatography/electrospray ionization (LC-MS/MS, Bruker Esquire 3000 plus Ion Trap). The LC-MS/MS analysis was carried out as described [13]. Briefly, peptides were separated by chromatography on a 75 μm Χ 15 cm PepMap nanocolumn (LC Packings) at a flow rate of 250 nL/min using a gradient increasing from 5 to 95% acetonitrile in 0.1% formic acid over 60 min. The column effluent was sprayed directly into the ion trap which was set to scan the m/z range from 300 to 1,500 in positive ion mode, capturing MS and MS2 data automatically.

### Protein identification criteria

Data captured by LC-MS/MS were matched using the Mascot algorithm (Matrix Science, UK, http://www.matrixscience.com) to an in-house nonredundant annotated protein database for *P. falciparum *(3D7 strain) containing 5,460 sequences (4,087,115 residues). Carbamidomethylation of cysteine and oxidation of methionine residues were considered as fixed and variable modifications respectively and a single missed cleavage was permitted. For LC-MS/MS data, peptide mass tolerance was at ± 1 Da, MS/MS ion mass tolerance was set at ± 0.5 Da and charge states (+2, +3) were taken into account. The probability that the observed match between the experimental data and the database sequence was a random event was set to a significance threshold rate of *p *< 0.001 and by using a decoy database the maximum false discovery rate under these conditions was estimated to be less than 1%. The minimum required peptide length was six amino acids. Proteins with at least two peptides (at least one thereof uniquely assigned to the respective protein sequence) were considered identified.

## Results

The main aim of this study was to develop an improved work-flow to identify plasmodial proteins of interest using knowledge of their mass and isoelectric point. This strategy was dependent on the capacity of protein OFFGEL™ to deliver sharply focussed proteins distributed at the appropriate pI value in either 12 or 24 fractions. This was assessed as follows.

### Influence of HDP on the quality of protein focussing

**Figure 1:**
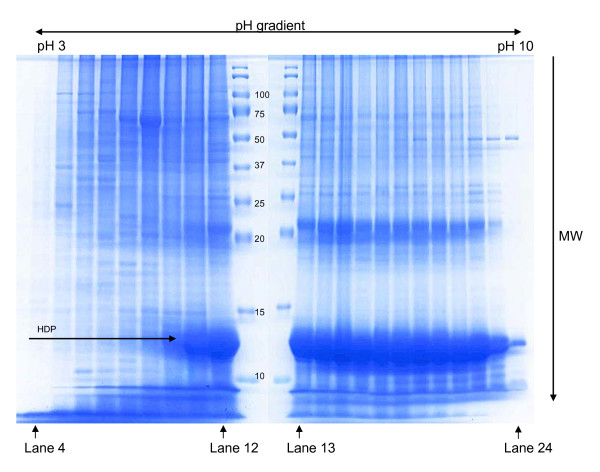
**OFFGEL™ analysis of proteins extracted from *P. falciparum *using chaotropes**. Large amounts of HDP, which hinder isoelectric focussing, can be seen in the large, intensely staining smear at approximately 12 kDa which localizes from lanes 10 to 23.

For analysis by mass spectrometry, the conventional method for the extraction of protein from *P. falciparum *is based on the use of chaotropes and reducing agents. The combination of urea, thiourea and dithiothreitol (DTT) leads to extensive protein denaturation, and ultimately protein solubilization [17]. However, when such lysates were prepared from *P. falciparum *and fractionated on the OFFGEL™ apparatus, HDP were seen to make up a large part of the total material. As displayed in Figure 1, this was distributed across a wide range of pI values (14 of the 24 fractions). Moreover the failure of the HDP material to focus successfully had a profoundly negative impact on the separation of the plasmodial proteins. Thus, it seems to have acted as a "carrier" for other proteins with an isoelectric point in the range of fractions 10 to 24, and as a consequence little focussing seems to have occurred, leaving the same plasmodial proteins distributed across many of these fractions. In contrast, in fractions 4 to 8, where there are no detectable HDP, the plasmodial proteins have focussed much more successfully, often to a single fraction, demonstrating the potential of the OFFGEL™ approach.

### Freeze-thaw lysis avoids HDP extraction

**Figure 2:**
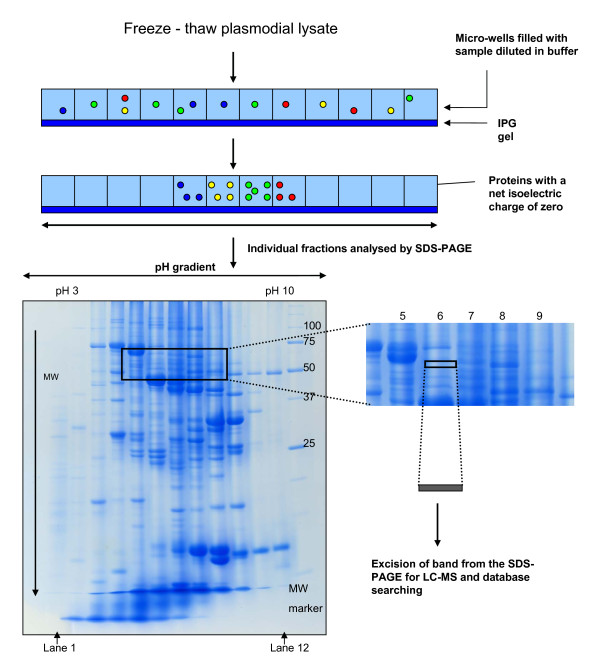
**The protein-centric OFFGEL™ strategy**. Proteins in a freeze-thaw lysate were separated by OFFGEL™ electrophoresis in the first dimension followed by SDS-PAGE for the second dimension. Bands in the gel area between the masses of 50 to 70 kDa and lanes 5 to 9 were analysed by mass spectrometry. Between ten and twelve 1 mm thick bands were analysed for each lane between these masses.

The preliminary observations described above suggested to us that whilst OFFGEL™ separation at the protein level had significant potential, this could not be properly delivered from extracts containing high levels of HDP. Further investigation therefore sought to identify a parasite lysis procedure that could release cytosolic parasite proteins free from contamination with HDP. This was ultimately achieved using five cycles of freezing and thawing in deionized water. After acetone precipitation, resuspension in OFFGEL™ sample buffer and overnight OFFGEL™ fractionation, SDS-PAGE analysis of the resulting fractions (Figure 2) revealed little evidence of HDP and, presumably as a consequence of this, excellent focussing of plasmodial proteins across the entire range of pI values.

### Analysis of isoelectric focussing of proteins using OFFGEL™ electrophoresis

**Figure 3:**
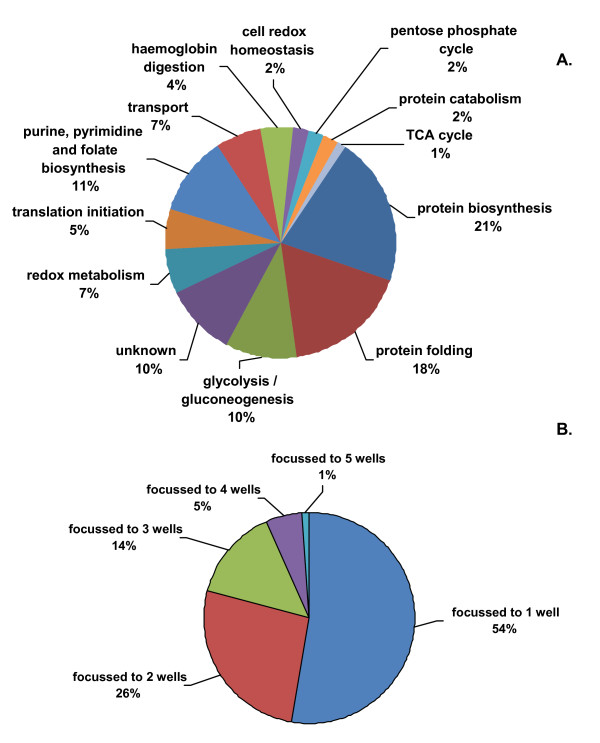
**Functional identification and classification of the 91 unique *P. falciparum *proteins identified by mass spectrometry**. **A**. A total of 91 unique proteins were identified by OFFGEL™ electrophoresis and mass spectrometry. Classifications were based on annotated data from Uniprot [19] and the Malaria metabolic pathways resource [20]. **B**. Of the proteins identified, 80% were focussed to no more than 2 fractions while 54% achieve "perfect" focussing, (i.e. proteins that focus to 1 fraction only).

The manufacturer of the OFFGEL™ apparatus recommends a protein load of between 50 μg and 5 mg. Hubner and colleagues found that for peptide fractionation, loading 250 μg of peptides gave the most peptide identifications by LC-MS without compromising focusing quality, which was assessed by the percentage of peptide sequences detected in a single OFFGEL™ fraction [18]. In order to facilitate the coupling of an orthogonal SDS-PAGE separation to the isoelectric focussing step, 1 mg of protein was loaded onto the OFFGEL™ apparatus to allow for sample losses from the subsequent acetone precipitation and in-gel protein digestion steps. To assess the success of protein focussing, protein, precipitated from OFFGEL™ fractions, was anlaysed by 1D SDS-PAGE. Gel bands were excised from lanes loaded with samples derived from adjacent fractions 5, 6, 7, 8 and 9 between the protein mass ranges of 50 to 70 kDa (between 10 and 12 visible bands from each lane, Figure 2). This area was selected as it is a region of the gel where protein molecular weight and isoelectric point calculations indicated that the majority of target proteins; DHFR-TS, DHFS-FPGS, HPPK-DHPS, and SHMT should be located. After trypsin digestion and LC-MS/MS analysis, a total of 91 proteins were identified in this area of the gel and annotated according to UniProt [19] and the Malaria Metabolic Pathways resource [20] (Figure 3a and Additional File 1). The quality of protein focusing was then assessed. It was determined that 80% of proteins focussed to less than two OFFGEL™ fractions and 54% of proteins achieved "perfect focussing" (a given protein being identified in only one OFFGEL™ fraction) (Figure 3b). Only one protein, enolase, was found in all five fractions, but this is known to have at least seven different isoforms [21] which would explain its presence in multiple fractions.

### Validation of OFFGEL™ isoelectric protein focussing

**Figure 4:**
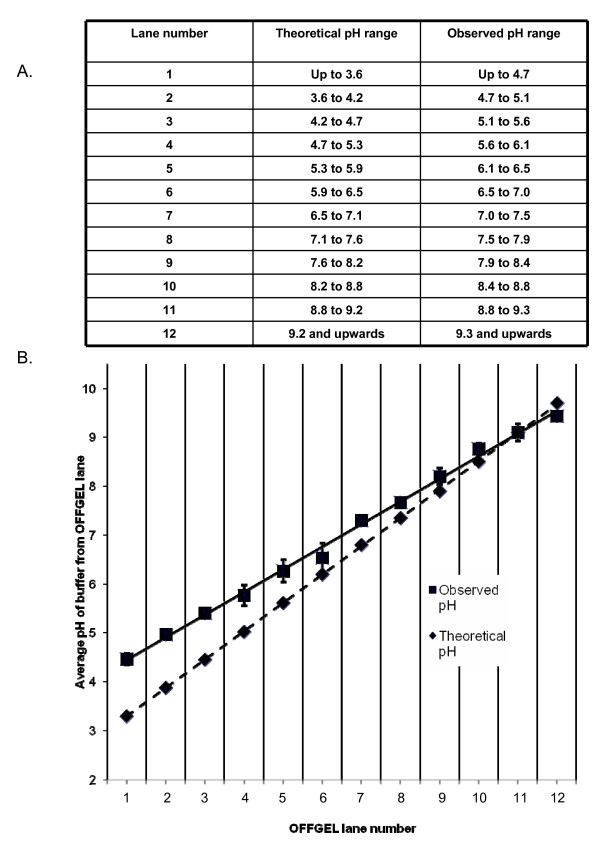
**Theoretical and observed pH ranges of individual OFFGEL™ fractions**. **A**. The theoretical pH range was calculated by dividing by 12 the 7 available pH units between pH 3 and 10 to give a value of approximately 0.6 pH units for the range covered by each fraction. The median value for each fraction was then plotted. The observed pH for each fraction was calculated by averaging the pH measured for equivalent fractions from three OFFGEL™ separations. **B**. Plots of the median theoretical and mean observed pH values for each OFFGEL™ fraction. The standard deviation for each individual OFFGEL™ fraction pH measurement is also displayed.

Commercially available IPG strips from GE Healthcare were used in validating the OFFGEL™ fractionation. These are strips of 13 cm in length containing an immobilized pH gradient ranging from pH 3 to 10. A frame consisting of 12 equal sized fractions was placed on top of the IPG strip. To assign pH ranges to the individual OFFGEL™ fractions, a "theoretical pH range" was constructed. The assumption was made that the distribution of immobilized ampholytes on the IPG strip comprised a linear gradient within the range from pH 3 to 10. Dividing the 7 available pH units by 12 gave a graduation of approximately 0.6 between each fraction. Therefore, fraction 1 had a theoretical pH range from 3 to 3.6, fraction 2 ranged from 3.6 to 4.2 and so on up to pH 10. This is displayed in Figure 4a. An "observed pH range" was also determined. For this, three OFFGEL™ fractionations were performed using only OFFGEL™ buffer as sample, and the pH of each fraction was directly measured and corresponding values averaged. This information is also displayed in Figure 4a. From graphical analysis of these data (Figure 4b), it is evident that the immobilized pH gradient measured after an OFFGEL™ separation is linear, but begins at approximately pH 4.2, and not pH 3 as described by the manufacturer. Thus, proteins with a pI 4.7 and lower are focussed to fraction 1, while proteins whose pI lies between 4.7 and 5.2 are focussed to fraction 2 and so on. It has been noted previously that the immobilized pH gradient in commercial IPG strips can vary both in length and position [18]. This may explain the distribution of the IPG gradient as determined by this study and is a phenomenon to be aware of when utilizing an isoelectric point-based identification approach. Table 1 further illustrates this point. Using the annotation information of the 91 proteins categorized in Figure 3, the mean and median isoelectric point of the proteins identified in each fraction was calculated. These show reasonable agreement with the theoretically expected values but agree rather less well with pH ranges observed in the earlier experiments. Importantly however, the sequence coverage of proteins separated and identified using OFFGEL™ was excellent, with an average sequence coverage of 25% (Table 1).

**Table 1:** Protein isoelectric points and sequence coverage of the 91 unique proteins identified in this study

	Lane 5 (n = 24)	Lane 6 (n = 31)	Lane 7 (n = 34)	Lane 8 (n = 42)	Lane 9 (n = 38)
**Mean pI**	6.2 ± 1.1	6.4 ± 0.9	6.7 ± 0.9	6.7 ± 1.0	7.3 ± 0.8

**Median pI**	5.7	6.3	6.5	6.7	7.2

**Theoretical pH range**	5.3 to 5.9	5.9 to 6.5	6.5 to 7.1	7.1 to 7.6	7.6 to 8.2

**Observed pH range**	6.1 to 6.5	6.5 to 7.0	7.0 to 7.5	7.5 to 7.9	7.9 to 8.4

**Mean peptide coverage (%)**	24.5 ± 15.3	23.1 ± 12.6	21.3 ± 9.8	19.6 ± 12.0	35.8 ± 14.8

**Median peptide coverage (%)**	23	24	21	17	34

Bands from 5 lanes were excised between the 50 and 70 kDa region of the SDS-PAGE gel as displayed in Figure 2. Isoelectric points of proteins identified in each fraction were obtained from UNIPROT [19] and the mean and median values of these were calculated. The theoretical and observed pH ranges are as described in Figure 3a. The percentage peptide coverage of each protein identified in a particular fraction was used to calculate the mean and median values for peptide coverage of all proteins localized to that fraction where n = number of proteins identified.

### Identification of folate enzymes using OFFGEL™ fractionation and mass spectrometry

Theoretical masses and isoelectric point data for the enzymes of the folate pathway were obtained using UniProt [19]. These data were used to determine their expected positions within the SDS-PAGE gel following OFFGEL™ fractionation. To increase the probability of identifying the enzymes of interest, a mass position of plus and minus 10% of a particular enzyme's theoretical mass was used. Similarly, for isoelectric point, the lanes adjacent to that predicted to contain the protein (using the observed pH ranges) were also excised and subjected to analysis by mass spectrometry. By applying a significance threshold rate of *p *< 0.001 against a decoy database, the false discovery rate for the identified folate proteins was estimated to be less than 1%. Of the eight experimentally validated enzymes of the malarial folate pathway, six were detected in this study (Table 2). HPPK-DHPS and DHFR-TS are bifunctional so that a statistically significant identification to either protein domain meant that, by definition, the bifunctional enzyme had been identified.

**Table 2:** Proteins of the folate pathway identified in this study.

Name	PlasmoDB identifier	Average sequence coverage (%)	Mass (kDa)	pI	False discovery rate (%)
**PTPS**	PFF1360w	31	20280	5.97	0.58

**DHFR-TS**	PFD0830w	5	72660	6.86	0.3

**HPPK-DHPS**	PF08_0095	25	84062	6.73	0.24

**SHMT**	PFL1720w	28	49749	8.19	0

For each protein the observed protein molecular mass and isoelectric point corresponds to the theoretical values as obtained from UNIPROT. The percentage false discovery rate was calculated using a decoy database and the peptide homology or identity threshold set at p < 0.001.

### Identification of GTPCI and DHFS-FPGS by western blotting

**Figure 5:**
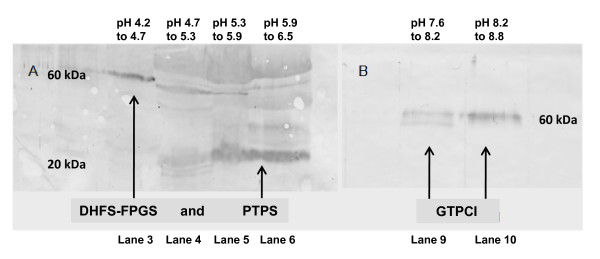
**Western blot analysis**. **A**. SDS-PAGE gel of OFFGEL™ fractions probed with anti-DHFS-FPGS. **B**. SDS-PAGE gel of OFFGEL™ fractions probed with anti-GTPCI. Anti-PTPS was used as a positive control after blotting and probing for DHFS-FPGS. The isoelectric point and molecular mass of both DHFS-FPGS and GTPCI differ significantly from their theoretical values. DHFS-FPGS would be expected to be identified from lane 6 at molecular mass 60 kDa, yet western blotting analysis places it in lane 3. GTPCI has a theoretical mass of 46 kDa and would be expected to be identified from lane 12, yet this analysis identifies it as being found in lanes 9 and 10 and at mass 60 kDa

The full complement of folate pathway enzymes was not identified by mass spectrometry in this study. Of the two proteins that were not identified, DHFS-FPGS has a theoretical isoelectric point of 6.25 based on its sequence, while that for GTPCI is estimated to be 9.4. On the SDS-PAGE gel, this would place DHFS-FPGS in lane 6 (pH range 5.9 to 6.5) and GTPCI in lane 12 (pH range 9.2 and upwards) with molecular masses of 60 and 46 kDa respectively. However, these enzymes were not identified at those positions. Their presence in the gel was however confirmed by western blotting (Figures 5a and 5b). As a positive control, an antibody to the protein previously identified by mass spectrometry as PTPS (pI 6.0, MW 20 kDa) was also used to probe a filter prepared by transfer of proteins from an SDS-PAGE analysis of an OFFGEL™ separation. As revealed in this experiment, its location confirmed the mass spectrometry results, with its position on the SDS-PAGE gel corresponding to its calculated molecular mass and isoelectric point. However, western blotting also localized DHFS-FPGS to lane 3 (pH range of 4.2 to 4.7) on the same filter, and GTPCI was determined to be in both lanes 9 and 10 (pH range 7.6 to 8.8) of a second filter. The observed mass for DHFS-FPGS was estimated to be 60 kDa, similar to its theoretical mass, while GTPCI was estimated to have a mass also of approximately 60 kDa, which differs significantly from its theoretical mass of 46 kDa. Although it was thus apparent that the two unidentified proteins were not running as expected on OFFGEL™ and/or SDS-PAGE, further excision of bands in areas of the gel highlighted by western blotting followed by subsequent mass spectrometry still failed to identity these two enzymes. It is also noted that the western analysis reported here reveals the possible existence of at least two forms of GTPCI differing slightly in their mobility on SDS-PAGE,

## Discussion

Detection, after OFFGEL™ separation, of the full complement of folate enzymes from *P. falciparum *has been reported in this study. The presence of HPPK-DHPS, DHFR-TS, PTPS and SHMT could be experimentally verified by mass spectrometry. This represents a significant step forward from previous work reported by this laboratory, where it was only possible to confidently identify the most abundant of these enzymes, SHMT [2]. However, the presence of GTPCI and DHFS-FPGS could only be revealed using western blotting in these experiments indicating that their isoelectric points, and the molecular mass of the former, differed from theoretical data. The results obtained by mass spectrometry, at the least, were only achievable using a fractionation strategy that reduces sample complexity and thus improves identification. The presence of highly abundant material such as HDP and albumin from plasmodial culture medium is to the detriment of the collection of quality mass spectral data. HDP comprise a significant percentage of the extracted sample when a lysate is prepared in the conventional manner with chaotropes and reducing agents. Here, it is evident that an overabundance of HDP disturbs the separation of the other proteins during IEF, as clearly demonstrated in Figure 1. To circumvent the release of HDP into the lysate, a work-flow utilizing freeze-thaw extraction is described. Freeze thawing has been used successfully for the extraction of plasmodial cell proteins in the past [22]. It is believed to cause lysis through the disruption of cell membranes with the formation of ice crystals, leading to swelling of the cells and ultimately the rupture of cell membranes and release of cytosolic proteins. The data in Figure 2, Additional File 1 and Table 2 are a striking demonstration that the removal and/or fractionation of high-abundance components facilitates the separation and identification of proteins of mid- to low-abundance such as the folate enzymes.

This is the first in-depth investigation of the isoelectric focussing of proteins, rather than peptides, using OFFGEL™ electrophoresis. The OFFGEL™ fractionation of peptides is more frequently utilized, mostly for "shotgun" proteomics where the end result of either 12 or 24 fractions for mass spectrometric analysis generates a valuable reduction in sample complexity. The work reported here demonstrates that there is also a case to be made for protein OFFGEL™ however. The quality of focussed proteins obtained using this system was comparable with focussing quality achieved for peptides [18]. A peptide load of 100 mg allowed the successful focussing of 86% of identified peptide sequences to two fractions. Figure 3b shows that a load of 1 mg protein lysate achieved almost the same degree of focussing quality (80%). While the sample here comprised cytosolic proteins, which would be expected to be amenable to solution-based isoelectric focussing, it cannot be ignored that significantly higher sample loads of protein compared to peptide can be successfully fractionated using OFFGEL™ separation. This may arise because, as seen in Figure 2, proteins are fairly evenly distributed across the majority of the pH range of the IPG strip, in comparison to peptide fractionation using OFFGEL™, where the majority of peptides focus to the acidic and basic ends of the strip [23]. Moreover, unlike peptide OFFGEL™ fractionation, it is the MW and pI parameters of the intact protein that determine its behaviour during OFFGEL™ separation and, as this work demonstrates, these can be useful identifiers of where to search for a given protein. The reduction of sample complexity is the primary aim in both protein and peptide OFFGEL™ fractionation, and for this, their localization to fractions as predicted by isoelectric point is not essential, yet it is pleasing to note the success in this respect, as displayed in the identification of folate biosynthesis proteins in Table 2.

The difficulties encountered in identifying GTPCI and DHFS-FPGS by mass spectrometry reveal the possible shortcomings in using theoretical isoelectric point data to identify the pH range within which a protein will localize. Western blotting (Figures 5a and 5b) reveals that the isoelectric points of DHFS-FPGS and GTPCI differ significantly from their theoretical values. This difference may be due to a number of factors including modification of the protein, either by splicing or by post-translational modification. While the average isoelectric points of proteins within each fraction show good agreement with the theoretical pH ranges across those fractions, the spread of the isoelectric points of the proteins is quite large (Table 1). This indicates either that the calculation methods from which the theoretical isoelectric points are derived are not wholly accurate, or that some of the proteins are modified in such a way as to subtly alter their predicted isoelectric points. As long as a reduction in sample complexity is the main objective, this will not matter. However, for a targeted identification of proteins, as was the objective here, it is a factor that must be considered. As a minimum, including a reasonable margin of error when excising a region of the gel for analysis by mass spectrometry is recommended.

Approximately 50 slices were excised and processed from the representative sample area of the SDS-PAGE gel to identify the 91 unique proteins as summarized in Figure 3a. If this is extrapolated to cover the whole area of the gel, it is estimated that around 2000 proteins should be identifiable. In practice, processing this number of samples is only achievable with automation. For global proteomic studies however, the most obvious strategy is to omit the SDS-PAGE step, to proteolytically digest each of the 12 OFFGEL™ protein fractions, and then perform a second OFFGEL™ separation, this time at the peptide level, to decrease complexity further, as numerous other studies have done.

## Conclusions

The data presented here demonstrate the capability of OFFGEL™ to successfully and reproducibly separate milligram quantities of protein into 12 fractions Moreover, they strongly suggest that full orthogonal separation using both protein and peptide level fractionation should be achievable. This, combined with the improved lysis procedure that is also described here, will facilitate the analysis of the *P. falciparum *proteome to a depth that was previously unattainable.

## Competing interests

The authors declare that they have no competing interests.

## Authors' contributions

RO'C carried out all of the experimental work and drafted the manuscript. JEH and PFGS conceived the study and revised the manuscript. All authors were involved in experimental design, data interpretation and analysis and have read and approved the final manuscript.

## Supplementary Material

Additional file 1 **Details of 91 *P. falciparum *proteins identified in this study**. Names, properties and locations in which each of the 91 proteins identified in this study were found. Click here for file
